# Water-Soluble Palladium Complexes with 1,10-Phenanthroline—Synthetic Aspects, Crystal Structure, DNA-Binding and In Vitro Antibacterial Evaluation

**DOI:** 10.3390/molecules31030576

**Published:** 2026-02-06

**Authors:** Marina A. Uvarova, Ilya A. Yakushev, Nina A. Kasyanenko, Natalia A. Komolkina, Noura Hilal, Igor L. Eremenko

**Affiliations:** 1 N.S. Kurnakov Institute of General and Inorganic Chemistry, Russian Academy of Sciences, Leninsky Prosp. 31, GSP-1, 119991 Moscow, Russia; ilya.yakushev@igic.ras.ru (I.A.Y.); ilerem@igic.ras.ru (I.L.E.); 2 Department of Molecular Biophysics and Polymer Physics, Faculty of Physics, Saint Petersburg State University, Universitetskaya Emb., 7–9, 199034 Saint Petersburg, Russia; nkasyanenko@mail.ru (N.A.K.); st069690@student.spbu.ru (N.A.K.);

**Keywords:** palladium complexes, DNA binding, crystal structure, 1,10-phenanthroline, antibacterial activity, synthesis

## Abstract

Obtaining water-soluble palladium complexes capable of interacting with DNA is an important synthetic task in medicinal chemistry. The interaction of [Pd(phen)(OAc)_2_] (phen = 1,10-phenanthroline) with pivalic acid (^t^BuCOOH) and trifluoromethanesulfonic acid (HOTf) leads to the formation of the molecular complex [Pd(phen)(OOC^t^Bu)_2_] (**1**) and the ionic complex [Pd(phen)(H_2_O)_2_]Otf_2_ (**2**), respectively. Complex **1** is highly soluble in water and stable in solution for 48 h. When complex 2 is boiled in water, it undergoes hydrolysis to form the binuclear hydroxo-bridged complex [Pd_2_(phen)_2_(μ-OH)_2_]Otf_2_ (**3**). According to X-ray diffraction data, the crystal lattices of **1**–**3** are stabilized by numerous intermolecular hydrogen bonds and π-π stacking interactions. The interaction of **1** and **2** with DNA in vitro (in 0.005 M NaCl solution) was studied using UV spectroscopy, low-gradient viscometry, and DNA melting analysis. It was shown that both compounds interact with DNA, and the binding is accompanied by the intercalation of the phenanthroline ligand at low concentrations in the DNA solution. An increase in their concentration leads to an alternative binding mode—palladium–DNA interaction causes a decrease in the DNA molecular coil size due to electrostatic interaction and/or palladium coordination to DNA bases. The difference between the binding of compounds **1** and **2** to DNA is that **2** can coordinate to N-bases, unlike complex **1**. The antibacterial properties of the complexes have been studied in vitro against *E. coli*, *P. aeruginosa*, and *S. aureus.*

## 1. Introduction

The development of new drugs based on metal complexes began to progress rapidly after the discovery of cisplatin, a platinum-based complex for the treatment of cancer [1]. Platinum drugs are a crucial component of cancer chemotherapy; for example, cisplatin is involved in the treatment of various cancers, including sarcomas, soft tissue and bone cancers [2,3]. Due to the serious toxicity and side effects of platinum drugs, there is a need to search for new biologically active metal complexes. The coordination structure and geometry of palladium (II) are very similar to those of platinum (II), so various palladium(II) complexes have been proposed as drug candidates due to their excellent antitumor and antibacterial activity [4,5]. An example of such a compound is the water-soluble palladium complex padeliporphine, used in vascular targeted photochemotherapy for the treatment of prostate cancer [6]. It was also investigated that palladium(II) complexes containing thiosemicarbazone ligands showed higher activity against *E. Coli* and *S. aureus* than free ligands [7,8]. It has also been studied that the coordination of antibiotics to palladium makes them active against the virulent *Mycobacterium tuberculosis strain H37R*, *E. coli*, *S. aureus*, and *P. streptococcus* [9,10,11]. One of the ways to reduce the toxicity and increase the stability of palladium complexes is the introduction of chelated ligands such as phenanthroline and bipyridyl into their structure [12,13,14,15]. It was previously shown that among palladium complexes with various N-donor ligands, complexes with phenanthroline were active against ovarian adenocarcinoma cancer cells with very low toxicity to healthy cells, and also showed antibacterial activity against *M. smegmatis* [16]. Many studies show that 1.10-phenanthroline significantly enhances the antibacterial activity of metal complexes [17,18,19,20,21]. The mechanism of the toxic action of platinum-based drugs is their covalent binding to DNA [22,23,24]. It is assumed that palladium complexes will also be able to bind to DNA, and the presence of a planar aromatic chelate ligand in the metal complex, such as phenanthroline, makes it possible to integrate into the DNA structure by the type of intercalation [25,26,27]. Along with phenanthroline, the nature of the anion can play a crucial role in the binding of the palladium complex to DNA, since weak donor groups can be easily displaced from the complex by DNA N-bases, forming a coordination bond [28].

An important criterion for the selection of biologically active compounds is their water solubility and stability in solution, which are largely determined by the nature of the anion [29,30,31,32,33]. To study the effect of the nature of the anion in complexes [Pd(phen)X_2_], we have selected two anions that are very different in nature. Triflate anion, the anion of strong trifluoromethanesulfonic acid, is hydrophilic and can easily be displaced from the coordination sphere of palladium by DNA bases, forming covalent binding. In contrast, the pivalate anion contains a hydrophobic tert-butyl group and is strongly bound to metal even in the presence of N-donor bases, and the resulting planar complex can intercalate with DNA. Herein, we report a synthetic route to different types of palladium complexes—the molecular complex [Pd(phen)(OOC^t^Bu)_2_] (**1**), the ionic complexes [Pd(phen)(H_2_O)_2_]Otf_2_ (**2**), and [Pd_2_(phen)_2_(μ-OH)_2_]Otf_2_ (**3**)—along with studies of their crystal structures, DNA-binding abilities, and antibacterial activity against Gram-negative *Escherichia coli* and *Pseudomonas aeruginosa* and Gram-positive *Staphylococcus aureus*. The primary aim of this work is to investigate the influence of the anion in mononuclear palladium(II) complexes with 1,10-phenanthroline on solubility, stability, mode of DNA interaction, and antibacterial activity.

## 2. Results

### 2.1. Synthesis of Compounds ***1***–***3***

**Scheme 1:**
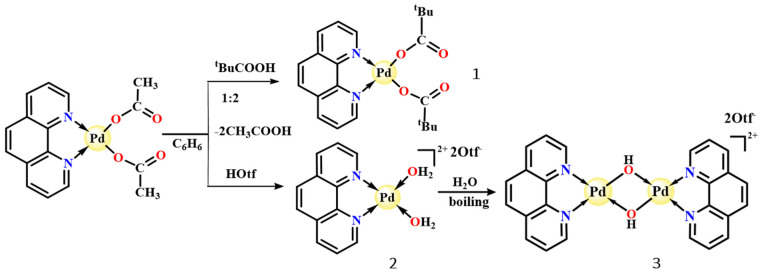
Synthesis of **1**–**3**.

The mononuclear complexes were synthesized by anion exchange reactions between previously known palladium(II) complex [Pd(phen)(OAc)_2_] [34] with excess of pivalic or trifluoromethansulfonic acid in benzene. It was found that in the case of weak pivalic acid, a molecular complex of the composition [Pd(phen)(OOC^t^Bu)_2_] (**1**) is formed. Whereas, when strong trifluoromethanesulfonic acid is used, the triflate anion is replaced in the outer sphere, forming an ionic complex [Pd(phen)(H_2_O)_2_]Otf_2_ (**2**). (Scheme 1). The two water molecules coordinated to the palladium atom are explained by the use of non-dried solvents. A similar reaction in acetonitrile leads to a complex [Pd(phen)(MeCN)_2_]Otf_2_ [35]. Both complexes **1** and **2** were found to be readily soluble in water_._ Boiling an aqueous solution of complex **2** for 1 h leads to hydrolysis to form a binuclear hydroxybrided complex [Pd_2_(phen)_2_(OH)_2_]Otf_2_ (**3**). The same hydrolysis was observed for the complex [Pd(dien)H_2_O]^2+^, and also led to the formation of the hydroxo-bridged complexes [Pd_2_(dien)_2_(μ-OH)_2]_^2+^ [36]. Wimmer also describes in detail the process of hydrolysis of palladium and platinum complexes, leading to the formation of bridging hydroxide anions [37].

The structure of the complexes is confirmed by FTIR spectroscopy (Figures S1–S3).

The spectrum of complex **1** contains characteristic bands of the carbonyl group 1619 cm^−1^ (ν(C=O), 1478 cm^−1^ (νs(C–O)), and C–H fluctuations in tert-butyl substituents 1391 cm^−1.^ 1318 cm^−1^. Strong bands of CF_3_-groups of triflate anions are present in spectra—1255 cm^−1^, 1218 cm^−1^ in **2**, 1242 cm^−1^, 1159 cm^−1^ in **3**. Vibrations of the SO_3_ group of the triflate anion give strong bands of 1023 and 1022 cm^−1^ in **2** and **3**, respectively. Bands, corresponding to fluctuations in aromatic rings of phenanthroline, are present in **1**, **2,** and **3**. Complex **3** contains fluctuations in the OH groups at 3393 cm^−1^_._

### 2.2. Single-Crystal X-Ray Structure

**Figure 1:**
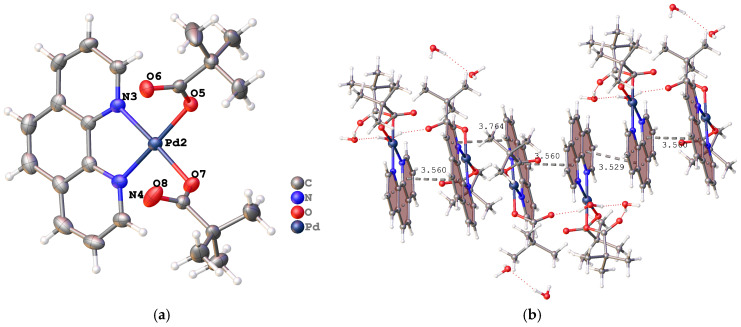
General view of compound **3** with thermal ellipsoids drawn at the 50% probability level (**a**) and a fragment of the crystal packing of **1** (**b**). The dotted line shows hydrogen bonds (red lines), π-π interactions (gray lines).

Compound **1** is a mononuclear complex in which the palladium atom is coordinated by two nitrogen atoms of the chelating phenanthroline ligand and two oxygen atoms from monodentately bound pivalate anions. The bulky tert-butyl substituents introduce steric strain, distorting the square-planar geometry of the palladium center (e.g., angle N(1)-Pd(1)-N(2) = 82.19(10)°, O(1)-Pd(1)-N(2) = 96.33(9)°). The main bond lengths are listed in Table 1. Complex 1 crystallizes with three solvate water molecules, which form hydrogen bonds with the free oxygen atoms of the carboxylate groups. The crystal packing is further stabilized by π-π stacking interactions between the phenanthroline fragments of neighboring molecules, generating one-dimensional chains along the crystallographic *a* axis (Figure 1a).

Thus, the molecular units are interconnected via numerous non-covalent interactions—O–H⋯O hydrogen bonds, π-π stacking, and C–H⋯π contacts—leading to the formation of supramolecular layers in the crystal structure (Figure 1b).

**Figure 2:**
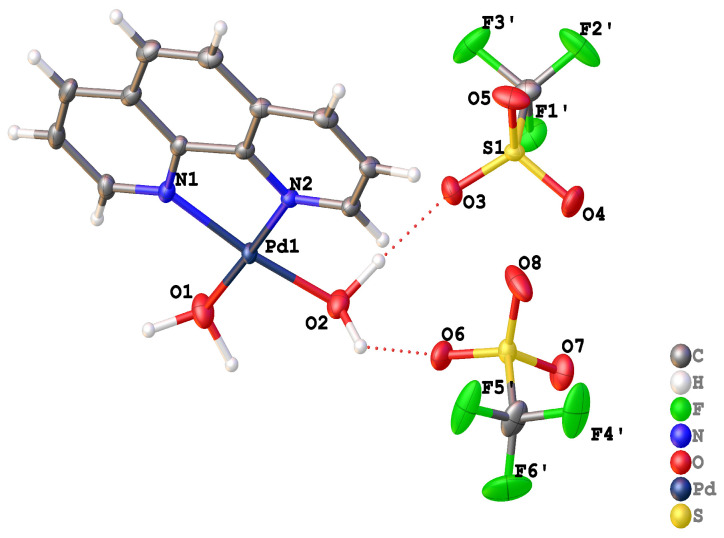
General view of compound **2** with thermal ellipsoids drawn at the 50% probability level.

Compound **2** is an ionic complex in which the palladium cation is coordinated by a chelating phenanthroline ligand and two water molecules (Figure 2). The palladium center adopts a distorted square-planar geometry: the angles N(1)–Pd(1)–N(2) and O(1)–Pd(1)–O(2) are reduced to 81.63° and 88.55°, respectively, while the angles O(1)–Pd(1)–N(1) and O(2)–Pd(1)–N(2) are increased to 94.61° and 94.47°, respectively. The two triflate anions reside in the outer sphere and are engaged in hydrogen-bonding interactions with the water molecules coordinated to palladium. A similar angular distortion was previously observed in the related complex [Pd(phen)(MeCN)_2_]Otf_2_ [35].

Complex **2** crystallizes with one benzene solvate molecule, which participates in a π-π stacking interaction with the aromatic rings of the phenanthroline ligand (centroid–centroid distance 3.807 Å, dihedral angle 1.313°). These intermolecular interactions lead to the formation of chains propagating along the crystallographic *b* axis. The coordination of water molecules to palladium(II) in the solid state is relatively uncommon [38,39].

**Figure 3:**
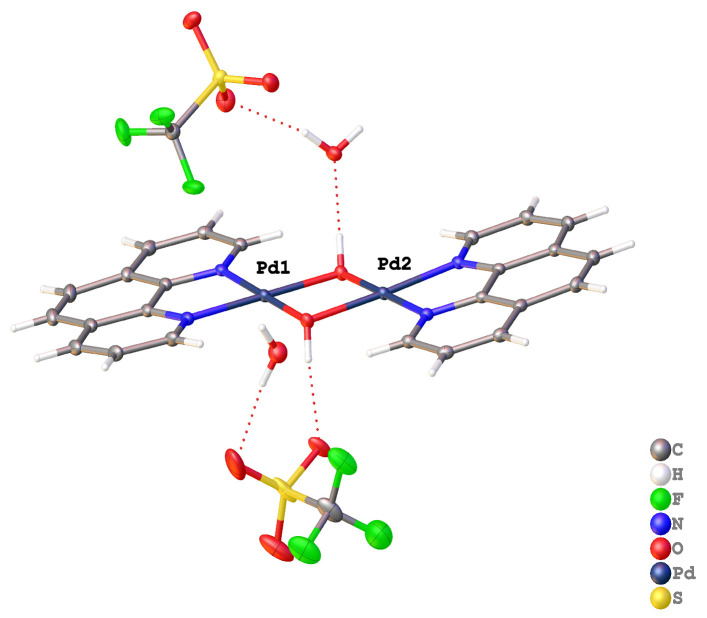
General view of compound **3** with thermal ellipsoids drawn at the 50% probability level. The dotted red lines show hydrogen bonds.

The hydrolysis of complex **2** yields the binuclear μ-hydroxo-bridged complex [Pd_2_(phen)_2_(OH)_2_]Otf_2_ (**3**). Compound **3** is an ionic complex whose cationic moiety comprises two palladium atoms at a non-bonding distance of Pd(1)···Pd(2) = 3.0018(7) Å, linked by two bridging hydroxide anions (Figure 3). Each palladium center is coordinated by a chelating phenanthroline ligand, forming a square-planar N_2_O_2_ coordination environment. This environment results in a nearly planar cationic unit, as evidenced by the small torsion angles [Pd1–O1–Pd2–O2 = 3.148° and N1–N2–N3–N4 = 1.864°]. The two triflate anions in the outer sphere form hydrogen bonds with the bridging hydroxide ligands and solvate water molecules. A similar formation of binuclear hydroxo-bridged palladium compounds containing an N_2_Pd(OH)_2_PdN_2_ core has been previously reported [40,41]. The previously described complex [Pd_2_bpy_2_(OH)_2_]Otf_2_ [42] has a very similar structure to complex **3**; however, the presence of an additional solvate water molecule in **3** significantly alters the hydrogen-bonding network and, consequently, the crystal packing.

**Figure 4:**
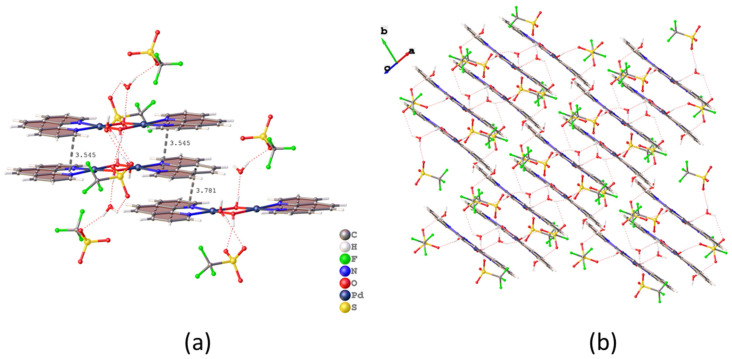
Fragment of π-π interactions (**a**) and crystal packaging (**b**) of **3**. The dotted line shows hydrogen bonds (red lines), π-π interactions (gray lines).

The crystal packing of **3** is stabilized by a number of intermolecular hydrogen bonds and π-π interactions between aromatic rings of phen, the distance between the centroids of PdN2C2 is 3.454 Å in pairs (Figure 4). Planar cations form layers due to intermolecular interactions in the crystal lattice.

### 2.3. DNA Interactions

**Figure 5:**
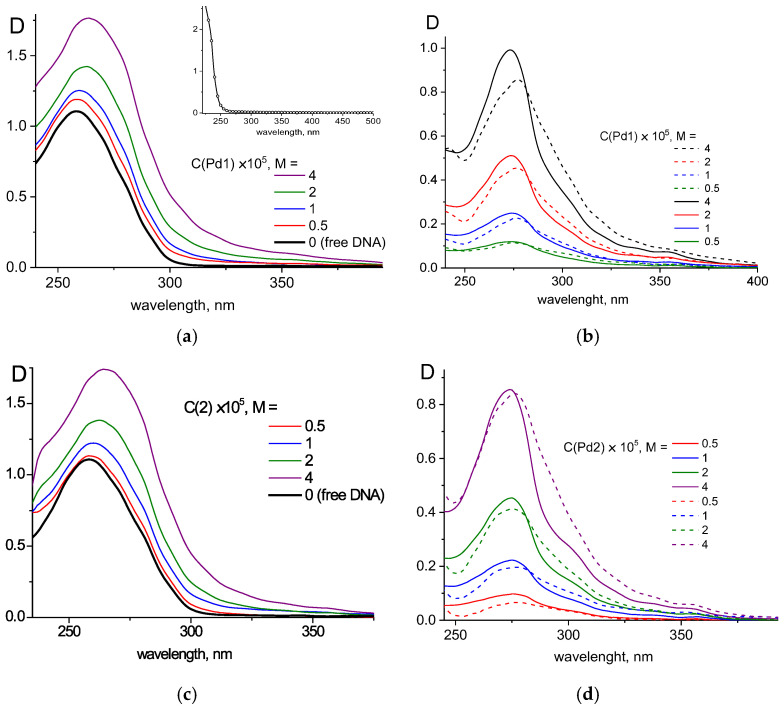
UV absorption spectra of free DNA and DNA complexes with compounds **1** (**a**) and compounds **2** (**c**) in 0.005 M NaCl with 10% of DMSO, insert to (**a**) shows DMSO absorption; the absorption of free **1** and **2** (solid lines) and their calculated spectra in complexes with DNA (dash lines) are shown in (**b**) and (**d**) respectively. Concentrations of **1** and **2** are shown near the lines.

The interaction of DNA with compounds **1** and **2** was studied using spectral and hydrodynamic methods. Solutions of compounds **1** and **2** in DMSO were transferred into aqueous solutions containing 0.005 M NaCl. The DMSO content in the studied solutions was kept constant at 10%. It has been shown that DMSO in a DNA solution does not alter the DNA conformation at concentrations C(DMSO) < 20% [43]. The contribution of DMSO to the measured absorbance is negligible at wavelengths λ > 250 nm (see inset in Figure 5a).

The absorption spectra of free DNA and DNA complexes with **1** are presented in Figure 5a. The experiment was performed at a constant DNA concentration and varying concentrations of compound **1**.

Both components involved in the interaction (DNA and the palladium compounds) absorb light within the same spectral region. Assuming that DNA absorption does not change upon binding with **1**, we calculated the absorption spectrum of the palladium compound within the complexes. These spectra differ from the spectra of the free compound in the absence of DNA (Figure 5b). Hypochromism and a red shift in the absorption band can be observed in the DNA complexes. Similar spectral changes were observed for DNA complexes with **2** (Figure 5c,d). The spectral data indicate binding of compounds **1** and **2** to DNA, with the chromophores of the palladium compounds being involved in this interaction.

**Figure 6:**
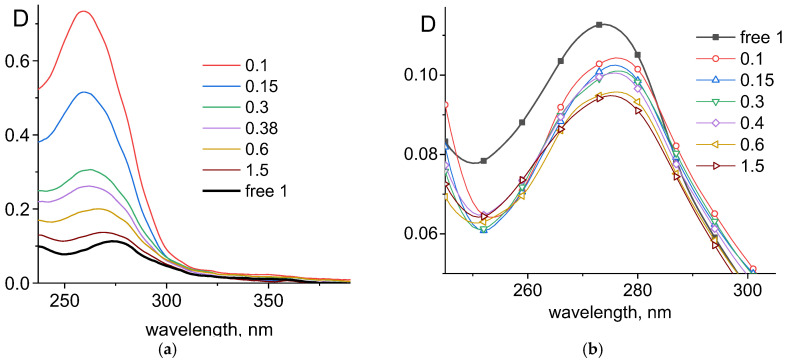

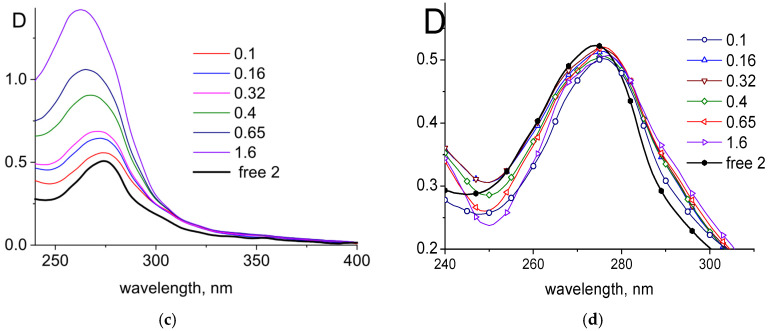
Absorption spectra of free **1** (**a**) and **2** (**c**) and their complexes with DNA in 0.005 M NaCl with 10% DMSO, the r values are shown near the lines; calculated spectra of **1** (**b**) and **2** (**d**) in complexes with DNA at constant concentrations of **1** and **2**.

In the subsequent experiment, solutions of palladium compounds **1** or **2** were used at a constant palladium concentration and varying DNA concentrations (Figure 6a,c). The calculated spectra of **1** (Figure 6b) and **2** (Figure 6d) in the complexes also show bathochromic shifts and hypochromism. The change in DNA concentration is expressed as a change in the **r** value, where **r** is the ratio of the molar concentration of compound **1** or **2** to the molar concentration of DNA base pairs.

The spectral changes observed during DNA binding with compounds **1** and **2** show some similarities (hypochromism, bathochromic shift). At the same time, the changes in the absorption of **1** upon binding are slightly more pronounced. It should be noted that the hypochromism observed in the calculated absorption bands of **1** and **2** in their DNA complexes could arise from subtracting a DNA absorption value higher than that in the actual complexes. However, significant hypochromism is atypical for DNA absorption itself. This suggests that the changes in the absorption of the palladium compounds’ chromophores in the complexes are likely attributable to their binding to DNA.

The absence of significant differences in the spectral changes observed upon binding of these compounds to DNA may indicate similarities in their binding modes. The main chromophores of the palladium compounds are phenanthroline ligands. Due to its planar structure, phenanthroline can intercalate between DNA base pairs. One of the key methods for confirming intercalation is viscometry.

**Figure 7:**
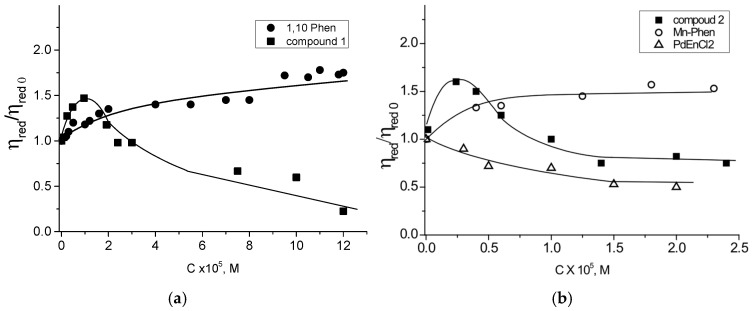
Dependence of the relative change in reduced viscosity of DNA solutions on concentrations of compound **1** (**a**) and **2** (**b**). Data for 1,10-phenanthroline (**a**) for Mnphen and PdEnCl_2_ (**b**) are also presented.

To test the hypothesis of phenanthroline intercalation, the reduced viscosity of DNA solutions was measured at constant DNA concentration and varying concentrations of **1** or **2**. Indeed, viscometry results support this hypothesis (see Figure 7a,b). It was shown that the reduced viscosity of DNA solutions in 5 mM NaCl initially increases with the concentration of either palladium compound (**1** or **2**), followed by a gradual decrease. This type of dependence indicates the existence of two distinct binding modes. The first mode predominates at low concentrations of **1** and **2** in the DNA solution. This binding is accompanied by an increase in solution viscosity, characteristic of intercalation. However, at higher concentrations of **1** and **2**, this increase is reversed, leading to a decrease in viscosity. A similar decrease in DNA solution viscosity was observed for its binding with a palladium coordination compound lacking phenanthroline, such as PdEnCl_2_ (see Figure 7b). We can therefore assume that a second binding mode begins to predominate at higher concentrations of compounds **1** and **2** in the DNA solution. Such viscosity changes can be attributed to the electrostatic shielding of the DNA phosphate groups upon binding of the palladium compounds. Such charge shielding occurs when complex ions bind to DNA, regardless of the specific binding type, including the formation of a coordination bond, as was observed for cisplatin following its aquation [44].

Thus, viscometry indicates similarities in the binding of **1** and **2** to DNA and suggests the possibility of phenanthroline ligand intercalation at low concentrations of the palladium compounds.

It is interesting to note the significant difference between the trends observed for compounds **1** and **2** and the data previously obtained for the manganese coordination compound Mn(phen)(OAc)_2_·2H_2_O, which contains a single 1,10-phenanthroline ligand [45]. It was shown that this manganese compound interacts with DNA through coordination of the manganese ion to the N7 atom of guanine in the DNA major groove, accompanied by intercalation of the 1,10-phenanthroline ligand. The structure of this manganese compound allows both binding types to occur simultaneously.

**Figure 8:**
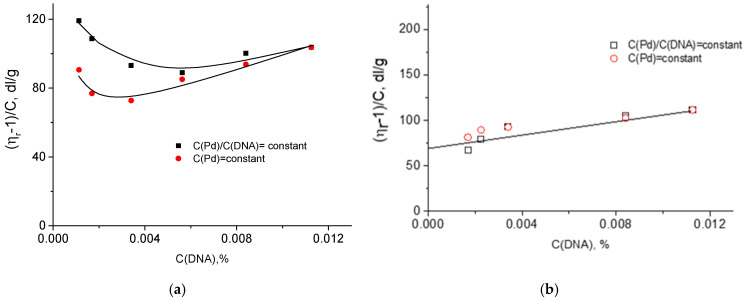
Dependence of the reduced viscosity of DNA solutions with compound **1** (**a**) and with compound **2** (**b**) on the concentration of DNA. The result of the experiment with two ways of dilution of stock solutions (solution with DNA complexes with compound **1** or with compound **2**) in 0.005 M NaCl. The dilutions of stock solutions at the highest DNA concentration with C(Pd) = const. and C(Pd)/C(DNA) = const. are presented.

The coordination of palladium to DNA bases can be assessed using a previously developed and validated experimental procedure based on different methods of diluting the stock solution containing pre-formed complexes [46]. Figure 8b shows that diluting the stock solution with 0.005 M NaCl (which maintains a constant C(Pd)/C(DNA) ratio) and diluting it with a palladium compound solution in 0.005 M NaCl at the same concentration as in the stock solution (which maintains a constant C(Pd) while decreasing the DNA concentration) yield completely identical trends for the DNA complexes with compound **2**. This result indicates that intentionally disrupting the equilibrium between bound and free palladium compounds in the DNA solution does not alter the DNA-**2** complexes during dilution. Such a result is observed only after the coordination of metal from metallocomplexes to DNA [46].

A different result was obtained for DNA complexes with compound **1** (Figure 8a). Different trends are observed for the two dilution methods. This shows that for compound **1**, palladium does not coordinate to DNA bases. This experiment highlights a fundamental difference in the DNA-binding mechanisms of **1** and **2**.

**Figure 9:**
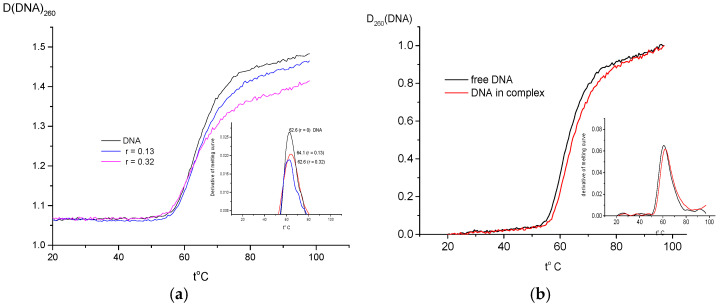
Melting curves of free DNA in 0.005 M NaCl and DNA in complexes with compound **2** (**a**) at different r values and **1** (**b**) (with 10% DMSO in all solutions), and their derivatives.

The DNA melting results obtained in solutions containing 10% DMSO may be influenced by the effect of DMSO on the stability of the macromolecule’s secondary structure upon heating. However, preliminary data indicate that the complexation of compound **2** with DNA at r = 0.13 (a condition favoring phenanthroline intercalation) slightly stabilizes the DNA structure, as is typically observed for intercalative binding (Figure 9). A similar stabilizing effect is observed for compound **1**. These results suggest that the binding of both compounds **1** and **2** to DNA does not destabilize its secondary structure.

At the same time, the second binding mode differs between the two compounds. Compound **1** appears to interact with DNA primarily through electrostatic forces, while its phenanthroline ligands may form stacked structures. In contrast, compound **2** is capable of forming coordination bonds (Pd–DNA).

### 2.4. Antibacterial Activity

The antibacterial activity of compounds **1** and **2** was determined in vitro against Gram-negative bacteria *Escherichia coli* ATCC 25922, *Pseudomonas aeruginosa* ATCC 27853 and one Gram-positive bacterium *Staphylococcus aureus* ATCC 29213. All the results obtained for the in vitro bioactivity of the compounds studied were compared to the activity of the standard antibiotic Tetracycline. The results of antibacterial activity **1**, **2** are shown in Table 2.

The study results indicate that the palladium complexes exhibit moderate antibacterial activity against the tested Gram-negative bacterial strains. The inhibition zones observed for **1** and **2** were smaller than those for tetracycline. The larger inhibition zone of complex **2** compared to **1** suggests its superior activity, which may be attributed to its ability to coordinate to DNA. Table 2 also shows that the complexes are less effective at suppressing bacterial growth than free phenanthroline. This is noteworthy, as the incorporation of a heavy metal ion typically enhances antibacterial effects [17]. At the same time, the Gram-positive bacterium *S. aureus* showed resistance to the studied complexes. This lack of activity against *S. aureus* could indicate an interaction with the bacterial membrane, given that membrane structure is a primary distinguishing feature between Gram-positive and Gram-negative bacteria.

It was previously shown that palladium complexes of a similar structure exhibit moderate activity against the Gram-positive strain *Mycobacterium smegmatis* and good activity against ovarian adenocarcinoma cells [16]. Furthermore, copper(II) phenanthroline complexes have demonstrated in vitro activity against *E. coli* and *S. aureus* comparable to that of standard antibiotics [47]. Alper Tolga Kolak et al. also studied a series of 3d-metal complexes (Co, Ni, Cu, Zn) with 1,10-phenanthroline and pyridine-2,5-dicarboxylate anions (Hpydc), observing moderate activity against *S. aureus* and very low activity against *E. coli* [48]. Thus, the antibacterial activity of the complexes appears to be determined not only by the presence of the phenanthroline ligand but also by the nature of the metal ion and the ancillary ligands. This indicates that, in addition to DNA interactions, the complexes likely affect other biological targets.

## 3. Conclusions

Water-soluble mononuclear palladium complexes with 1,10-phenanthroline and anions of pivalic acid and trifluoromethanesulfonic acid were synthesized and characterized. The nature of the anion significantly influences the structure and behavior of the complexes. The molecular complex [Pd(phen)(OOC^t^Bu)_2_] (**1**) is highly soluble in water and stable for a long time in an aqueous solution. In contrast, the ion complex [Pd(phen)(H_2_O)_2_]Otf_2_ (**2**) is stable in water at room temperature but undergoes hydrolysis upon boiling for one hour to form the binuclear hydroxo-bridged complex [Pd_2_(phen)_2_(OH)_2_]Otf_2_ (**3**). Spectroscopy (UV) and viscometry show that complexes **1** and **2** exhibit two binding modes, depending on their concentrations in DNA solutions. At low concentrations of complexes **1** and **2** in DNA solutions, intercalation of 1,10-phenanthroline is observed. At higher concentrations, complex **2** interacts with DNA via Pd coordination to DNA bases, while complex **1** binds to DNA via electrostatic interactions. Antibacterial evaluation showed that the complexes possess moderate activity against the Gram-negative bacteria *E. coli* and *P. aeruginosa.*

## 4. Materials and Methods

### 4.1. General Details

Commercial reagents and solvents were used for the synthesis: Pivalic acid (^t^BuCOOH), 99% Acros, trifluoromethansulfonic acid (HOtf, 99% Acros), benzene (≥99%). [PhenPd(OAc)_2_] was prepared by methodic described in [34].

IR spectra were recorded in the 400–4000 cm^−1^ region using a Spectrum-65 Perkin Elmer FT-IR spectrometer (PerkinElmer, Waltham, MA, USA). Microprobe analyses were carried out using a Carlo Erba EA 1108 Series CHN Elemental Analyze (Carlo Erba Instruments, Cornaredo, Italy) (Center of Collective Use of IGIC RAS). The UV-vis spectra were obtained using a Shimadzu UV-2600 spectrophotometer (Panreac, Barcelona, Spain), in the range of 220–400 nm. The stability of the complexes in aqueous solution was monitored by measuring the spectra of the sample (50 mM) for 48 h at room temperature. The ^1^H NMR spectra of solutions of the studied compounds in CD_3_CN were recorded on a QOne AS400 Quantum-I Plus (QOneTec, Dietlikon, Switzerland) spectrometer operating at 400.1 MHz, using an internal deuterium lock. Tetramethylsilane was used as a reference.

### 4.2. Synthesis of Complexes ***1***–***3***

*Synthesis of [Pd(phen)(OOC^t^Bu)_2_].* (**1**) Pivalic acid 0.1 g (1 mmol) was added to a solution of 0.1 g (0.25 mmol) of [Pd(phen)(OAc)_2_] in 10 mL of benzene and stirred at room temperature for 1 h. The resulting yellow compound was recrystallized from water. The obtained yellow crystals were separated from the mother liquor by decantation and dried in air. The yield of **1** was 0.92 g (76%). Anal. calc. C_22_H_29.77_N_2_O_5.89_Pd: C 50.54, H 5.74, N 15.36. Found: C 50.59, H 5.25, N 15.65. FT-IR (ATR, ν/cm^−1^): 2951 s, 2865 w, 1619 s,1517 w, 1478 m, 1425 m, 1391 m, 1318 s, 1209 s, 1147 w, 1029 w, 884 w, 846 s, 779 w, 714 s, 605 m, 641 m, and 439 m.

*Synthesis of [Pd(phen)(H_2_O)_2_]otf_2_.* (**2**) Trifluoromethansulfonic acid (0.1 mL, 1 mmol) was added to a suspension of 0.1 g (0.25 mmol) of [Pd(phen)(OAc)_2_] in 10 mL of benzene and stirred at room temperature for 1 h. 5 mL of THF was added to the resulting yellow solution, which was kept at +5 °C for a day. The resulting yellow crystals were separated from the mother liquor by decantation and dried in air. The obtained crystals were separated from the mother liquor by decantation. The yield was 0.12 g (80%). Anal. calc. C_17_H_15_F_6_N_2_O_8_PdS_2_: C 30.85, H 2.03, N 4.36. Found: C 30.95, H 2.29, N 4.25. FT-IR (ATR), ν/cm^−1^: 3457 s, 3098 w, 3064 w, 2943 w, 1717 w, 1585 w, 1522 w, 1431 w, 1255 s, 1218 s, 1149 s, 1023 s, 864 m, 807 w, 754 w, 717 s, 629 s, 568 m, 568 m, 512 s, and 448 w.

*Synthesis of [Pd_2_(phen)_2_(OH)_2_]otf_2_.* (**3**) 0.06 g (0.1 mmol) of complex **2** was boiled in water for 1 h. The yellow solution was left to cool to room temperature. The formed yellow crystals were separated from the mother liquor by decantation. The yield was 0.012 g (32%). Anal. calc. C_26_H_20.55_ F_6_N_4_O_9.28_ Pd_2_S_2_: C 35.76, H 4.42, N 6.36. Found: C 35.92, H 4.52, N 6.09. FT-IR (ATR), ν/cm^−1^: 3393 w, 3067 w, 1603 w, 1520 w, 1242 s, 1159 s, 1022 s, 845 m, 778 w, 711 s, 630 s, 548 m, and 511 s.

### 4.3. Antibacterial Activity of ***1***, ***2***

The antibacterial activity of the complex was estimated by the standard agar diffusion test method. Nutrient Agar was used as a medium with necessary modifications [49]. The antibacterial activity was tested against Gram-negative *Escherichia coli* ATCC 25922, *Pseudomonas aeruginosa* ATCC 27853 and one Gram-positive bacterium, *Staphylococcus aureus ATCC 29213* and the results were compared with the standard drug Tetracycline. In a laminar flow chamber, 20 mL of the molten agar medium was poured into a sterile Petri dish. Then, the paper disks impregnated with the solutions of the ligands and complexes in concentrations of 100, 50, and 25 μg/disk were placed on the surface of the media inoculated with the microorganisms. The culture was incubated for 24 h at +37 °C. The diameter of the zone of inhibition growth around the paper disk impregnated with the compound was determined. The tests were evaluated in triplicate. Results were reported as means ± standard errors (SE) calculated using MS Excel Statistical Analysis tool pack.

### 4.4. X-Ray Studies

The experimental data for compound **1**, **2** was obtained using Bruker D8 Venture diffractometer (Bruker, Bill Ricard, MA, USA) (Mo *K*α radiation, λ = 0.71073 Å, at 100 and 150 K, respectively, Center for Collective Use of the Kurnakov Institute RAS (Moscow, Russia)); the single-crystal X-ray data for complex **3** were collected using synchrotron X-ray radiation at the “Belok” beamline [50] of the Kurchatov Synchrotron Radiation Source (National Research Center “Kurchatov Institute”, Moscow, Russian Federation) in the ϕ-scan mode with the Rayonix SX165 CCD detector, λ = 0.75172 Å. The raw data for **1** and **2** were indexed and integrated with the APEX3 program suite [51]. Experimental intensities were corrected for absorption effects using SADABS [52]; the raw data for **3** were treated with the XDS data reduction program [53].

The crystal structures **1**–**3** were solved by direct methods [54] and refined by the full-matrix least-squares on *F*^2^ using the OLEX2 structural data visualization and analysis program suite [55]. All non-hydrogen atoms are refined with anisotropic thermal parameters, except low occupied oxygen atoms positions of water molecules in **1**. In the refinement of disordered moieties in structures **1**–**3**, restraints were used (SHELX instructions SADI, RIGU, SIMU, ISOR). All C–H hydrogen atoms were placed in the calculated positions and refined using the riding model with dependent isotropic thermal parameters with U_iso_(H) = 1.5U_eq_(C) for the methyl groups and with U_iso_(H) = 1.2U_eq_(C) for other hydrogen atoms; H-bonded hydrogen atoms of water generally were derived from Fourier synthesis map and refined freely or with dependent thermal parameters (U_iso_(H) = 1.5U_eq_(O)). The crystallographic data and structure refinement details for **1**–**3** are given in Table 3. CCDC entries *2516144-2516146* contain the supplementary crystallographic data for this paper. These data are provided free of charge by The Cambridge Crystallographic Data Centre: https://www.ccdc.cam.ac.uk/structures/, accessed on 3 February 2026.

### 4.5. DNA-Binding

High-molecular calf thymus DNA (Sigma Aldrich, St. Louis, MO, USA) with a molecular mass of M = 11 × 10^6^ as determined by the value of intrinsic viscosity of DNA [η] in 0.15 M NaCl from the formula [η] = 6.9 × 10^−4^ M^0.7^ was used.

*Spectral measurements*, including the study of DNA melting in complexes, were performed using an SF-2000 spectrophotometer (OKB Spectr, Saint-Petersburg, Russia) and a Specord 200 Plus (Analytic Jena, Jena, Germany).

*Low-gradient viscometry* was used to determine the molecular mass of DNA and to examine DNA conformation when interacting with compounds under study. The relative viscosity of DNA solutions, η_r_ = η/η_0_ (where η and η_0_ are the solution and the solvent viscosity, respectively), was measured at different velocity gradients g in the range of g = (0.5 ÷ 2) s^−1^. These conditions ensured the correct determination of the solution viscosity without the need to extrapolate the gradient dependence of viscosity to a zero gradient. The absence of gradient dependence was observed for all systems under study. The contribution to the measured η_r_ value of the other components in the solution, including salt and palladium complex compounds, was negligible. The reduced viscosity of solutions η_red_ = (η_r_ −1)/C was calculated, where C is the concentration of DNA. A low-gradient rotational viscometer was used. The principle of the viscometer was described in the article [56].

## Figures and Tables

**Table 1:** Selected bond lengths (Å).

	1	2	3
Pd–N	2.007(2), 2.013(2)	2.028(2), 2.039(2)	1.989(3)– 2.001(4)
Pd–O	1.991(2), 2.000(2)	2.041(2), 2.047(2)	2.000(3)– 2.016(3)

**Table 2:** The results of antibacterial activity.

	*E. coli* ATCC 25922			*P.aeruginosa* ATCC 27853			*S. aureus* ATCC 29213
C, µg/disk	25	50	100	25	50	100	50c
Compounds	The zone of inhibition, mm						
**1**	0	8 ± 0.3	11 ± 0.3	0	8 ± 0.6	15 ± 0.3	0
**2**	0	16 ± 0.6	20 ± 0.3	0	18 ± 0.6	20 ± 0.6	0
phen	11 ± 0.3	26 ± 0.3	30 ± 0.6	10 ± 0.3	19 ± 0.5	25 ± 0.3	0
[Cu(phen)(cnge)(NO_3_)_2_] [47]	-	23	-		0		-
[Co(Hpydc)_2_(phen)] [48]	-	7	8	-	-	-	8
[Ni(pydc)(phen)] [48]	-	0	0	-	-	-	0
[Cu(pydc)(phen)] [48]	-	12	20	-	-	-	18
[Zn(pydc)(phen)] [48]	-	0	-	-	-	-	13
Tetracycline	24	33	38	20	32	36	30

The diameter of the paper disk is 6 mm, phen-1,10 phenanthroline.

**Table 3:** Selected crystal data and parameters for structure refinement of **1**–**3**.

Parameters	Complex		
	1	2	3
Empirical formula	C_22_H_29.77_N_2_O_5.89_Pd	C_17_H_15_F_6_N_2_O_8_PdS_2_	C_26_H_20.55_ F_6_N_4_O_9.28_ Pd_2_S_2_
Temperature	100	150	100
Formula weight	522.83	659.83	928.42
Crystal system	Monoclinic	Triclinic	Triclinic
Space group	*P*2_1_/*c*	*P*-1	*P*-1
*a*(Å)	15.6964(4)	9.8525(10)	10.208(2)
*b*(Å)	17.5237(5)	11.5246(11)	12.9181(1)
*c*(Å)	18.4810(5)	11.7725(14)	13.0272(18)
*α*(°)	90°	110.522(3)	76.155(9)
β(°)	113.9370(9)°	107.931(3)°	72.322(19)°
γ(°)	90°	97.829(4)	68.224(3)°
V (Å^3^)	4646.2(2)	1145.5(2)	1504.3(4)
Z	8	2	2
*D*_calc._(g cm^3^)	1.495	1.913	2.050
μ(Mo*K*α) (cm^−1^)	837	1089	1659
2θ_max_, deg.	1.835 to 26.782°	1.962 to 30.491	2.455 to 30.537
*F*(000)	2151	654	914
*R*_int_	0.0426	0.0275	0.0335
Reflections collected	58,369	19,075	30,607
Independent reflections	9902	6914	7647
Parameters	624	415	491
*GooF*	1.037	1.139	1.016
*R*_1_, *wR*_2_ (*I* > 2*σ*(*I*))	*R*_1_ = 0.0361, *wR*_2_ = 0.0691	*R*_1_ = 0.0355, *wR*_2_ = 0.0910	*R*_1_ = 0.0425, *wR*_2_ = 0.1087
Δ*ρ*_max_, *ρ*_min_ (e/Å^3^)	0.451 and −0.666	0.800 and −1.481	1.052 and −1.496

## Data Availability

The original contributions presented in this study are included in the article/Supplementary Material. Further inquiries can be directed to the corresponding author.

## Supplementary Materials

The following supporting information can be downloaded at https://www.mdpi.com/article/10.3390/molecules31030576/s1, Table S1. Parameters of hydrogen bonds in the crystals, Figure S1. UV-vis spectra in aqua solution, Table S2. Selected bond lengthsand angles, Figures S2–S4 FTIR spectra of **1**–**3**, Figure S5—1H NMR spectra.

## Abbreviations

The following abbreviations are used in this manuscript:

FTIR	Fourier Transform Infrared Spectroscopy
DNA	deoxyribonucleic acid
UV	Ultraviolet

## References

[1] Rosenberg B., Van Camp L., Krigas T. (1965). Inhibition of Cell Division in Escherichia coli by Electrolysis Products from a Platinum Electrode. Nature.

[2] Wang D., Lippard S. (2005). Cellular processing of platinum anticancer drugs. Nat. Rev. Drug Discov..

[3] Wheate N.J., Walker S., Craig G.E., Oun R. (2010). The status of platinum anticancer drugs in the clinic and in clinical trials. Dalton Trans..

[4] Santos J.A., Félix M.M., Martins C.B., Marques J., Marques M.P.M., de Carvalho L.A.B. (2025). Palladium (ii) anticancer agents: The intricate case of the Pd–spermine complex. Dalton Trans..

[5] Kumar N., Kaushal R., Awasthi P. (2025). Transition Metal Complexes as Antibacterial Agents: An Overview. J. Fluoresc..

[6] Bugaj A.M. (2016). Vascular targeted photochemotherapy using padoporfin and padeliporfin as a method of the focal treatment of localised prostate cancer-clinician’s insight. World J. Methodol..

[7] Briñez-Ortega E., Sánchez-Mora A., Macías M.A., Brandão P.F., Chaparro D., Morales-Morales D., Burgos A.E. (2025). Synthesis of Pd (II) and Ag (I) complexes with pyrazole thiosemicarbazone: Characterization of the molecular structure by DFT/X-ray diffraction, in vitro antibacterial activity and molecular docking study. J. Mol. Struct..

[8] Aly A.A., Abdallah E.M., Ahmed S.A., Rabee M.M., Fuhr O., Ibrahim M.A.A., Alzahrani H.A., Youssif B.G. (2024). Synthesis and characterization of new palladium (II) and silver (I) thiosemicarbazone derived by acenaphthenequinone complexes and their antimicrobial activity. Polyhedron.

[9] Silva R.T.C., Guidotti-Takeuchi M., Peixoto J.L.M., Demarqui F.M., Mori A.P., Dumont C.F., Ferreira G.R.A., Pereira G.d.M., Rossi D.A., Corbi P.P. (2023). New Palladium(II) Complexes Containing Methyl Gallate and Octyl Gallate: Effect against Mycobacterium tuberculosis and Campylobacter jejuni. Molecules.

[10] Vieira L.M.M., de Almeida M.V., Lourenço M.C.S., Bezerra F.A.F.M., Fontes A.P.S. (2009). Synthesis and antitubercular activity of palladium and platinum complexes with fluoroquinolones. Eur. J. Med. Chem..

[11] Guerra W., de Andrade Azevedo E., de Souza Monteiro A.R., Bucciarelli-Rodriguez M., Chartone-Souza E., Nascimento A.M.A., Fontes A.P.S., Le Moyec L., Pereira-Maia E.C. (2005). Synthesis, characterization, and antibacterial activity of three palladium(II) complexes of tetracyclines. J. Inorg. Biochem..

[12] Yassin A.M., Elnouby M., El-Deeb N.M., Hafez E.E. (2016). Tungsten oxide nanoplates; the novelty in targeting metalloproteinase-7 gene in both cervix and colon cancer cells. App. Biochem. Biotech..

[13] Jaszczyszyn A., Gąsiorowski K., Świątek P., Malinka W., Cieślik-Boczula K., Petrus J., Czarnik-Matusewicz B. (2012). Chemical structure of phenothiazines and their biological activity. Pharmac. Rep..

[14] Albaqami F.F., Sahib A.S., Alharthy K.M., Altharawi A., Alshahrani M.Y., Jawad M.A., Suliman M., Ahmad I. (2025). A phenanthroline-based erbium (III) complex: Molecular docking, DNA/BSA-binding and biological evaluation. J. Biomol. Struct. Dyn..

[15] Marverti G., Gozzi G., Lauriola A., Ponterini G., Belluti S., Imbriano C., Costi M.P., D’Arca D. (2019). The 1,10-phenanthroline ligand enhances the antiproliferative activity of DNA-intercalating thiourea-Pd (II) and-Pt (II) complexes against cisplatin-sensitive and-resistant human ovarian cancer cell lines. Int. J. Molec. Sci..

[16] Uvarova M.A., Baravikov D.E., Dolgushin F.M., Aliev T.M., Titov K.O., Bekker O.B., Lashkin A.I., Malyants I.K., Shender V.O., Kiskin M.A. (2023). Antiproliferative and antimycobacterial effects of mononuclear palladium (II) complexes with N-heterocyclic ligands. Inorg. Chim. Acta.

[17] Viganor L., Howe O., McCarron P., McCann M., Devereux M. (2017). The antibacterial activity of metal complexes containing 1,10-phenanthroline: Potential as alternative therapeutics in the era of antibiotic resistance. Curr. Top. Med. Chem..

[18] Mengesha A.M. (2022). The study of antimicrobial activities of various transition metal mixed ligand complexes containing 1,10-phenanthroline with any other ligands. Int. J. Bioorg. Chem..

[19] Koshenskova K.A., Baravikov D.E., Nelyubina Y.V., Primakov P.V., Shender V.O., Maljants I.K., Lutsenko I.A. (2023). Copper (II) furancarboxylate complexes with 5-nitro-1,10-phenanthroline as promising biological agents. Russ. J. Coord. Chem..

[20] Uvarova M.A., Nikiforova M.E., Shmelev M.A., Aliev T.M., Metlin M.T., Taydakov I.V., Eremenko I.L. (2024). Lanthanide furoate complexes as promising systems with double effects–From suppression of mycobacteria to potential bioimaging. Inorg. Chim. Acta.

[21] O’Shaughnessy M., Hurley J., Dillon S.C., Herra C., McCarron P., McCann M., Devereux M., Howe O. (2023). Antibacterial activity of metal–phenanthroline complexes against multidrug-resistant Irish clinical isolates: A whole genome sequencing approach. J. Biol. Inorg. Chem..

[22] Sherman S.E., Lippard S.J. (1987). Structural aspects of platinum anticancer drug interactions with DNA. Chem. Rev..

[23] Sundquist W.I., Lippard S.J. (1990). The coordination chemistry of platinum anticancer drugs and related compounds with DNA. Coord. Chem. Rev..

[24] Pizarro A.M., Sadler P.J. (2009). Unusual DNA binding modes for metal anticancer complexes. Biochimie.

[25] Heydari M., Moghadam M.E., Tarlani A., Farhangian H. (2017). DNA as a target for anticancer phen-imidazole Pd(II) complexes. App. Biochem. Biotech..

[26] Mansouri-Torshizi H., Saeidifar M., Divsalar A., Saboury A.A. (2010). Interaction studies between a 1,10-phenanthroline adduct of palladium (II) dithiocarbamate anti-tumor complex and calf thymus DNA. A synthesis spectral and in-vitro study. Spectrochim. Acta Part A Mol. Biomol. Spectr..

[27] Barra C.V., Rocha F.V., Morel L., Gautier A., Garrido S.S., Mauro A.E., Netto A.V. (2016). DNA binding, topoisomerase inhibition and cytotoxicity of palladium (II) complexes with 1,10-phenanthroline and thioureas. Inorg. Chim. Acta.

[28] Binacchi F., Cirri D., Bimbi E., Busto N., Pratesi A., Biver T. (2025). Pd(II)/1,10-phenanthroline complexes bearing arene ligands: On the role of N-vs O-coordination to tune their cellular activity and binding ability towards DNA and RNA. J. Inorg. Biochem..

[29] Uvarova M.A., Lutsenko I.A., Shmelev M.A., Nefedov S.E., Bekker O.B., Lashkin A.I., Shender V.O., Eremenko I.L. (2024). Research of the influence of anions in complexes [CuPhen(Hpz)2 × 2] (X = CF3COO-, Otf-, Cl-) on the structure and bioactivity. New J. Chem..

[30] Zafar M.N., Masood S., Muhammad T.S.T., Dalebrook A.F., Nazar M.F., Malik F.P., Wright L.J. (2019). Synthesis, characterization and anti-cancer properties of water-soluble bis (PYE) pro-ligands and derived palladium (II) complexes. Dalton Trans..

[31] Abbehausen C., Sucena S.F., Lancellotti M., Heinrich T.A., Abrão E.P., Costa-Neto C.M., Formiga A.L., Corbi P.P. (2013). Synthesis, spectroscopic characterization, DFT studies, and antibacterial and antitumor activities of a novel water soluble Pd (II) complex with L-alliin. J. Mol. Struct..

[32] Kapdi A.R., Fairlamb I.J. (2014). Anti-cancer palladium complexes: A focus on PdX_2_L_2_, palladacycles and related complexes. Chem. Soc. Rev..

[33] İnci D., Aydın R., Vatan Ö., Şahin O., Çinkılıç N. (2019). Water-soluble binary and ternary palladium (II) complexes containing amino acids and intercalating ligands: Synthesis, characterization, biomolecular interactions and cytotoxicities. N. J. Chem..

[34] Milani B., Alessio E., Mestroni G., Sommazzi A., Garbassi F., Zangrando E., Randaccio L. (1994). Synthesis and characterization of monochelated carboxylatopalladium (II) complexes with nitrogen-donor chelating ligands. Crystal structures of diacetato (1,10-phenanthroline)-and diacetato (2,9-dimethyl-1,10-phenanthroline)-palladium (II). J. Chem. Soc. Dalton Trans..

[35] Uvarova M.A., Kushan E.V., Nefedov S. (2012). Synthesis and structure of palladium triflates with coordinated 3,5-dimethylpyrazole. Russ. J. Inorg. Chem..

[36] Bugarčić Ž.D., Petrović B.V., Jelić R. (2001). Hydrolysis of [Pt(dien)H_2_O]^2+^ and [Pd(dien)H_2_O]^2+^ complexes in water. Trans. Metal. Chem..

[37] Wimmer S., Castan P., Wimmer F.L., Johnson N.P. (1989). Preparation and interconversion of dimeric di-µ-hydroxo and tri-µ-hydroxo complexes of platinum (II) and palladium (II) with 2,2′-bipyridine and 1,10-phenanthroline. J. Chem. Soc. Dalton Trans..

[38] Qin Z., Jennings M.C., Puddephatt R.J. (2001). Self-assembly of polymer and sheet structures from palladium (II) complexes by hydrogen bonding between carboxamide substituents. Inorg. Chem..

[39] Gusev O.V., Kalsin A.M., Peterleitner M.G., Petrovskii P.V., Lyssenko K.A., Akhmedov N.G., Oberhauser W. (2002). Palladium (II) Complexes with 1,1′−Bis(diphenylphosphino) ferrocenes [Fe(η5-C5R4PPh_2_)_2_]^n+^ (dppf, R = H, n = 0; dppomf, R = Me, n = 0; dppomf+, R = Me, n = 1). Synthesis, Characterization, and Catalytic Activity in Ethene Methoxycarbonylation. Organometallics.

[40] Shen W.Z., Gupta D., Lippert B. (2005). Cyclic trimer versus head-tail dimer in metal-nucleobase complexes: Importance of relative orientation (syn, anti) of the metal entities and relevance as a metallaazacrown compound. Inorg. Chem..

[41] Conley N.R., Labios L.A., Pearson D.M., McCrory C.C.L., Waymouth R.M. (2007). Aerobic Alcohol Oxidation with Cationic Palladium Complexes: Insights into Catalyst Design and Decomposition. Organometallics.

[42] Adrian R.A., Benson R.E., Daniels L.M., Tiekink E., Walmsley J.A. (2006). Di-μ-hydroxo-bis [(2,2′-bipyridine)palladium(II)] trifluoro methane sulfonate. Acta Crystallogr. Sect. E Crystallogr. Commun..

[43] Kosmotynskaya Y.V., Bogdanov A.A., Kasyanenko N. (2005). Effect of dimethyl sulfoxide on DNA conformation in solution. Russ. J. Phys. Chem..

[44] Kasyanenko N., Aia E.E.F., Bogdanov A., Yakovlev K. (2002). Comparison of DNA Complexation with Antitumor Agent cis-DDP and Binuclear Bivalent Platinum Compound Containing Pyrazine. Mol. Biol..

[45] Kasyanenko N., Shatitsa M., Silanteva I., Demidov V., Komolkina N., Komolkin A. (2025). Comparative analysis of DNA interaction with Mn^2+^, 1,10-phenanthroline, mono- and binuclear complexes of manganese with 1,10-phenanthroline. Int. J. Biol. Macromol..

[46] Kasyanenko N., Qiushi Z., Bakulev V., Sokolov P., Yakovlev K. (2022). DNA Conformational Changes Induced by Its Interaction with Binuclear Platinum Complexes in Solution Indicate the Molecular Mechanism of Platinum Binding. Polymers.

[47] Aly S., El-Boraey H.A. (2019). Effect of gamma irradiation on spectral, XRD, SEM, DNA binding, molecular modling and antibacterial property of some (Z) N-(furan-2-yl) methylene)-2-(phenylamino) acetohydrazide metal (II) complexes. J. Mol. Struct..

[48] Colak A.T., Oztopcu-Vatan P., Colak F., Akduman D., Kabadere S., Uyar R. (2013). Syntheses, characterization, antimicrobial and cytotoxic activities of pyridine-2,5-dicarboxylate complexes with 1,10-phenanthroline. J. Trace Elem. Med. Biol..

[49] Perez C., Pauli M., Bazerque P. (1990). An antibiotic assay by the well agar method. Acta Biol. Med. Exp..

[50] Svetogorov R.D., Dorovatovskii P.V., Lazarenko V.A. (2020). Belok/XSA diffraction beamline for studying crystalline samples at Kurchatov Synchrotron Radiation Source. Cryst. Res. Technol..

[51] Bruker AXS Inc. (2016). APEX3, SAINT and SADABS.

[52] Krause L., Herbst-Irmer R., Sheldrick G.M., Stalke D. (2015). Comparison of silver and molybdenum microfocus X-ray sources for single-crystal structure determination. Appl. Crystallogr..

[53] Kabsch W. (2010). XDS. Biol. Crystallogr..

[54] Sheldrick G.M. (2015). SHELXT–Integrated space-group and crystal-structure determination. Found. Crystallogr..

[55] Dolomanov O.V., Bourhis L.J., Gildea R.J., Howard J.A., Puschmann H. (2009). OLEX2: A complete structure solution, refinement and analysis program. Appl. Crystallogr..

[56] Frisman E.V., Shchagina L.V., Vorobev V.I. (1965). A glass rotating viscometer. Biorheology.

