# Supplementary material for: Water-Soluble Palladium Complexes with 1,10-Phenanthroline—Synthetic Aspects, Crystal Structure, DNA-Binding and In Vitro Antibacterial Evaluation
Source: Molecules. 2026 Feb 6;31(3):576. doi: 10.3390/molecules31030576 (PMC12899409; doi:10.3390/molecules31030576)
Supplement: Supplementary file 1 [file molecules-31-00576-s001.zip › molecules-4087316-supplementary.pdf]

*Supporting Information*

# Water-Soluble Palladium Complexes with 1,10-phenanthroline—Synthetic Aspects, Crystal Structure, DNA-Binding and In Vitro Antibacterial Evaluation

**Marina A. Uvarova [1,*], Ilya A. Yakushev [1], Nina A. Kasyanenko [2], Natalia A. Komolkina [2], Noura Hilal [2] and Igor L. Eremenko [1]**

[1] N.S. Kurnakov Institute of General and Inorganic Chemistry, Russian Academy of Sciences, Leninsky Prosp. 31, GSP-1, Moscow 119991, Russia; ilya.yakushev@igic.ras.ru (I.A.Y.); ilerem@igic.ras.ru (I.L.E.)
[2] Department of Molecular Biophysics and Polymer Physics, Faculty of Physics, Saint Petersburg State University, Universitetskaya Emb., 7–9, Saint Petersburg 199034, Russia; nkasyanenko@mail.ru (N.A.K.); st069690@student.spbu.ru (N.A.K.)
* Correspondence: yak_marin@mail.ru

**Table S1**. Parameters of hydrogen bonds in the crystals of **1-3**

| D-H...A | d(D-H), Å | d(H...A), Å | d(D...A), Å | <(DHA), ° |
|---|---|---|---|---|
| Complex **1** | | | | |
| O(1W)-H(1WB)...O(6) | 0.87 | 1.94 | 2.783(3) | 161.7 |
| O(1W)-H(1WA)...O(2W) | 0.87 | 1.92 | 2.777(4) | 168.9 |
| O(2W)-H(2WA)...O(2) | 0.87 | 2.01 | 2.863(4) | 165.6 |
| O(2W)-H(2WB)...O(4)#1 | 0.87 | 1.92 | 2.771(3) | 165.4 |
| O(3W)-H(3WB)...O(8)#2 | 0.87 | 2.01 | 2.823(3) | 155.4 |
| O(3W)-H(3WA)...O(1W)#2 | 0.87 | 1.96 | 2.830(4) | 174.8 |
| O(4"^b)-H(4"A^b)...O(4) | 0.8405(1) | 2.906(3) | 3.236(3) | 105.69(5) |
| O(4"^b)-H(4"A^b)...O(2W)#2 | 0.8405(1) | 2.008(3) | 2.841(3) | 170.84(7) |
| O(4'^c)-H(4'E^c)...O(1W)#2 | 0.76(5) | 2.917(3) | 3.28(5) | 112(4) |
| O(4'^c)-H(4'E^c)...O(2W)#2 | 0.76(5) | 2.145(3) | 2.90(5) | 172(4) |
| O(4'^c)-H(4'D^c)...O(3W) | 0.86(5) | 1.879(3) | 2.73(5) | 172(3) |
| O(4W^a)-H(4WA^a)...O(3W) | 0.8396(1) | 2.191(3) | 2.922(3) | 145.56(7) |
| #1 x,-y+1/2,z+1/2 #2 x,-y+1/2,z-1/2 | | | | |
| Complex **2** | | | | |
| O(1)-H(1C)...O(4)#2 | 1.14(7) | 2.32(7) | 3.120(3) | 125(5) |
| O(1)-H(1B)...O(4)#3 | 1.13(7) | 1.91(7) | 3.029(3) | 170(5) |
| O(1)-H(1A)...O(8)#2 | 1.14(6) | 1.85(6) | 2.976(3) | 171(5) |
| O(2)-H(2B)...O(3) | 1.11(7) | 1.90(7) | 2.989(3) | 167(5) |
| O(2)-H(2A)...O(6) | 1.14(7) | 2.01(7) | 2.963(3) | 139(5) |
| O(2)-H(2C)...O(4)#3 | 1.11(6) | 2.20(6) | 3.092(3) | 135(4) |
| O(2)-H(2C)...O(7)#3 | 1.11(6) | 2.38(6) | 3.098(3) | 120(4) |

| | | | | |
|---|---|---|---|---|
| #1 -x+1,-y,-z+2 #2 x-1,y,z #3 -x+1,-y,-z+1 | | | | |
| Complex **3** | | | | |
| O(1W)-H(1WB)...O(5) | 0.85 | 2.06 | 2.883(6) | 164.4 |
| O(2W^c)-H(2WA^c)...O(2W^c)#1 | 0.8400(2) | 2.3325(4) | 2.8516(4) | 120.454(11) |
| O(1W)-H(1WA)...O(4)#2 | 0.85 | 2.25 | 2.981(5) | 144.6 |
| O(1W)-H(1WA)...F(6'^b)#3 | 0.85 | 2.72 | 3.233(17) | 120.3 |
| O(1W)-H(1WA)...O(7^a)#3 | 0.85 | 2.66 | 3.25(4) | 127.2 |
| O(2)-H(2)...O(6^a) | 0.843(3) | 2.106(6) | 2.908(7) | 158.7(3) |
| O(2)-H(2)...O(6'^b) | 0.843(3) | 1.934(4) | 2.726(5) | 156.1(3) |
| O(1)-H(1)...O(1W) | 0.841(3) | 1.872(3) | 2.703(4) | 168.9(2) |
| #1 -x+1,-y+2,-z+1 #2 -x+2,-y+1,-z #3 -x+1,-y+1,-z+1 | | | | |

**Table S2.** Selected bond lengths (Å) and angles (°).

| Bond | d, Å | Bond | d,Å | Bond | d,Å | Bond | d,Å |
|---|---|---|---|---|---|---|---|
| 1 | | 2 | | 3 | | | |
| Pd(1)-O(3) | 1.991(2) | Pd(1)-N(2) | 2.028(2) | Pd(1)-N(2) | 1.999(4) | Pd(2)-N(3) | 1.989(3) |
| Pd(1)-O(1) | 2.000(2) | Pd(1)-N(1) | 2.039(2) | Pd(1)-O(1) | 2.000(3) | Pd(2)-N(4) | 2.001(3) |
| Pd(1)-N(1) | 2.007(2) | Pd(1)-O(1) | 2.041(2) | Pd(1)-N(1) | 2.001(4) | Pd(2)-O(1) | 2.005(3) |
| Pd(1)-N(2) | 2.013(2) | Pd(1)-O(2) | 2.047(2) | Pd(1)-O(2) | 2.016(3) | Pd(2)-O(2) | 2.010(3) |
| | | | | | | | |
| Angle | ω(°) | Angle | ω(°) | Angle | ω(°) | Angle | ω(°) |
| O(3)-Pd(1)-O(1) | 89.63(9) | N(2)-Pd(1)-N(1) | 81.63(8) | N(2)-Pd(1)-N(1) | 82.49(15) | N(3)-Pd(2)-N(4) | 82.53(14) |
| O(3)-Pd(1)-N(1) | 91.90(9) | N(1)-Pd(1)-O(1) | 95.47(9) | O(1)-Pd(1)-N(1) | 97.15(13) | N(3)-Pd(2)-O(1) | 96.72(13) |
| O(1)-Pd(1)-N(2) | 96.33(9) | N(2)-Pd(1)-O(2) | 94.61(9) | N(2)-Pd(1)-O(2) | 97.24(14) | N(4)-Pd(2)-O(2) | 97.61(13) |
| N(1)-Pd(1)-N(2) | 82.19(10) | O(1)-Pd(1)-O(2) | 88.55(9) | O(1)-Pd(1)-O(2) | 83.13(12) | O(1)-Pd(2)-O(2) | 83.18(12) |

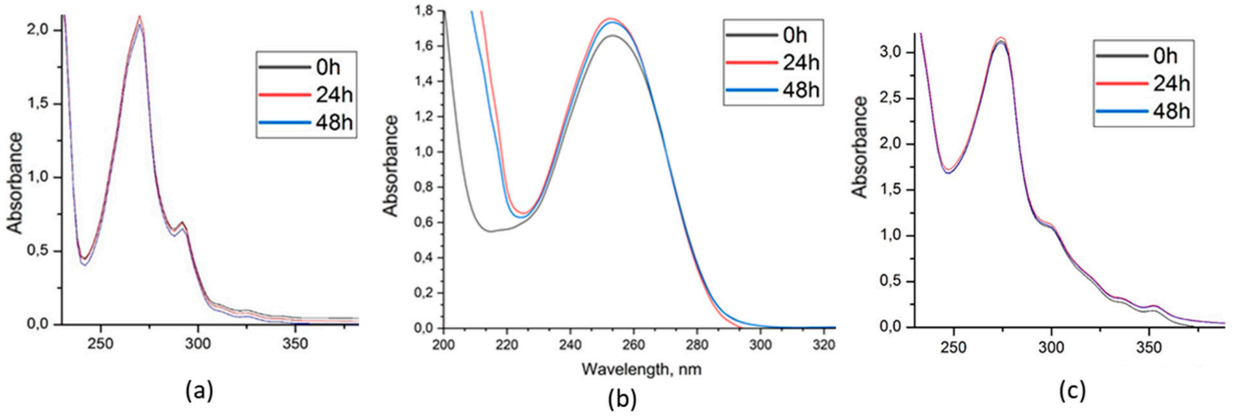


**Figure S1.** UV-vis spectra in aqua solution of 1(a), 2(b), 3(c).

The stability of the complexes in solutions was determined from the UV spectra of compounds 1-3 in DMSO solution. (Fig S2) Solution were registered every 24 h during 2 days). The intense bands (250-275 nm) correspond to *n*–π* and π–π* transitions in 1,10-phenanthroline. The band of charge transfer from metal to ligand was observed at 300-350 nm, however, it has low intensity. Solutions of complexes **1, 3** display similar changes in time. For complex 2, a slight shift in the spectrum is observed during the first 24 hours, however, prolonged exposure results in a stable solution without any changes at room temperature.

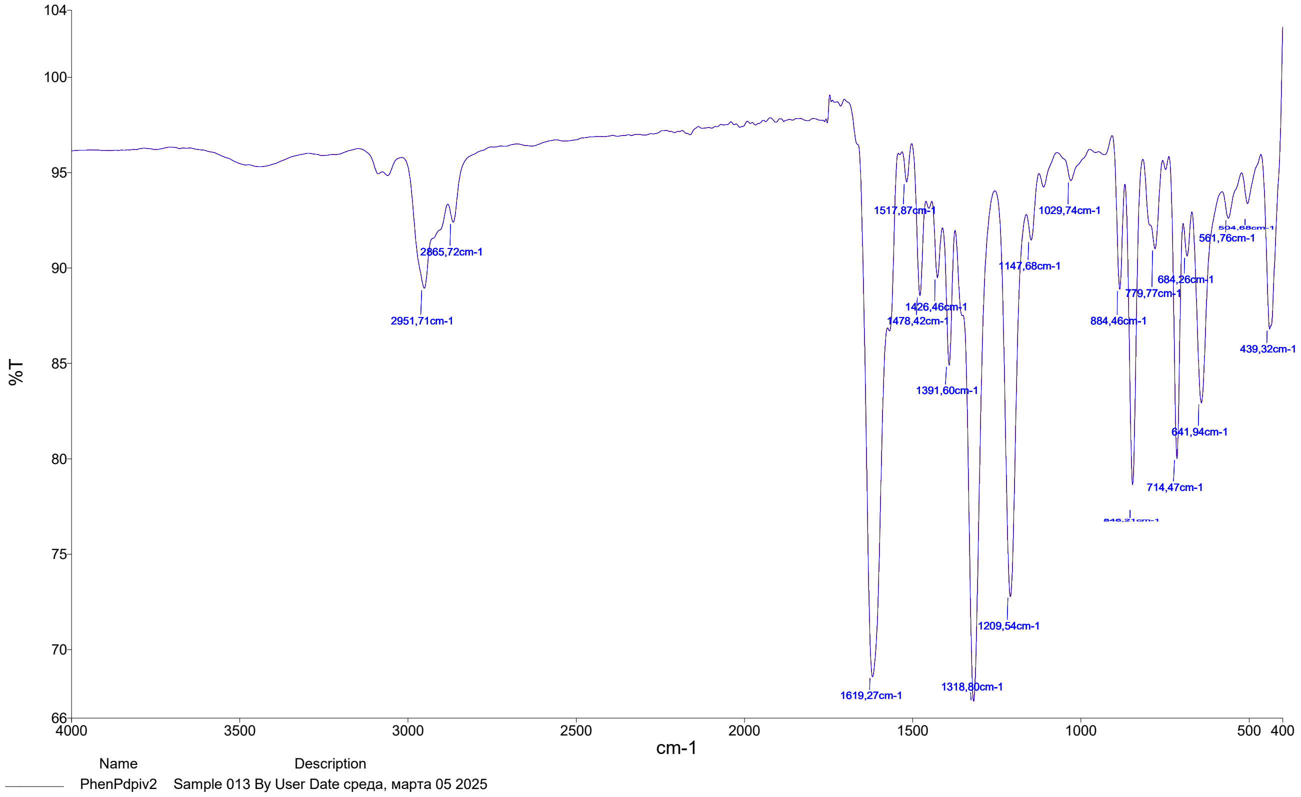


**Figure S2.** FTIR spectra of 1.

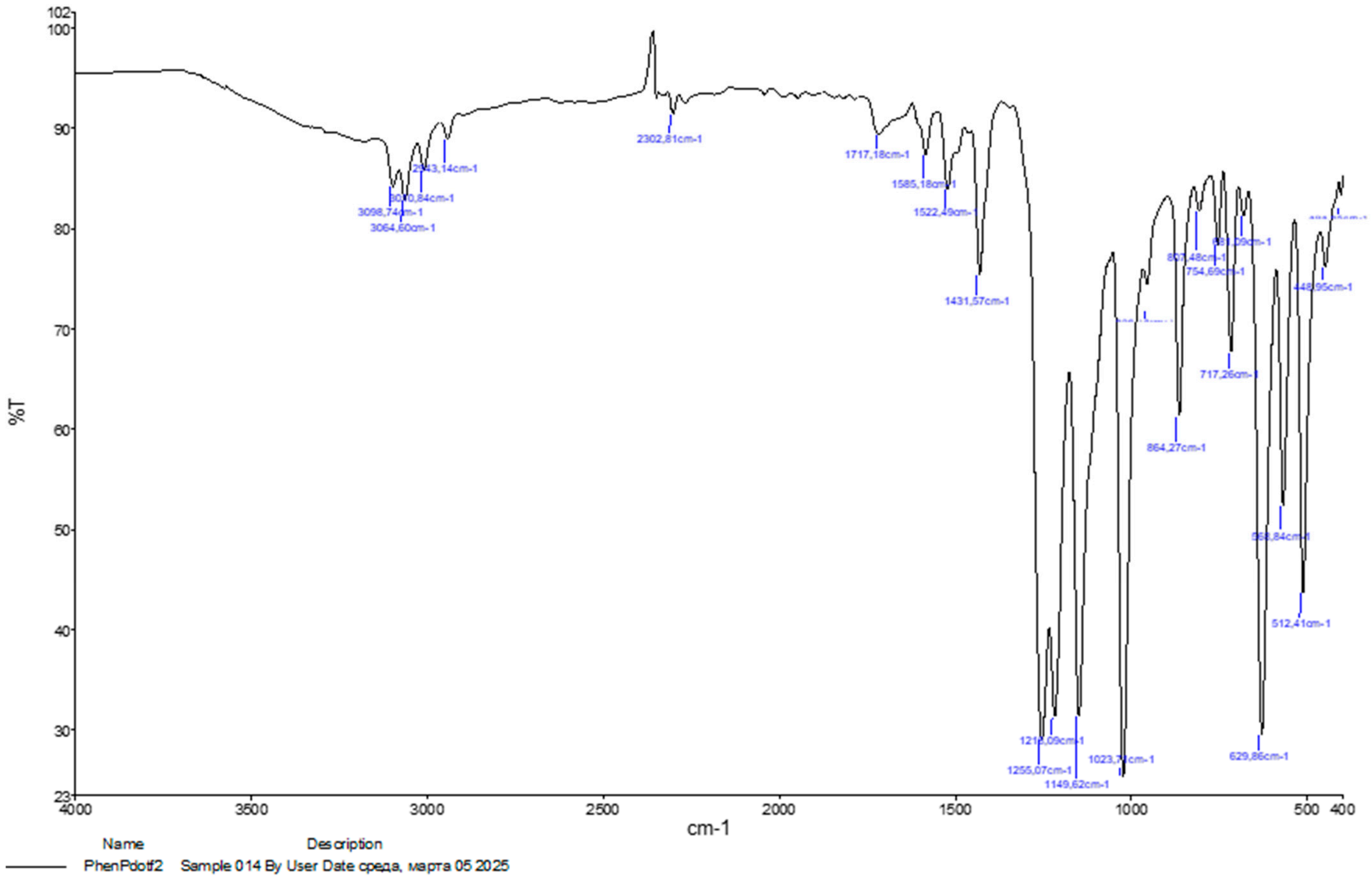


**Figure S3.** FTIR spectra of 2

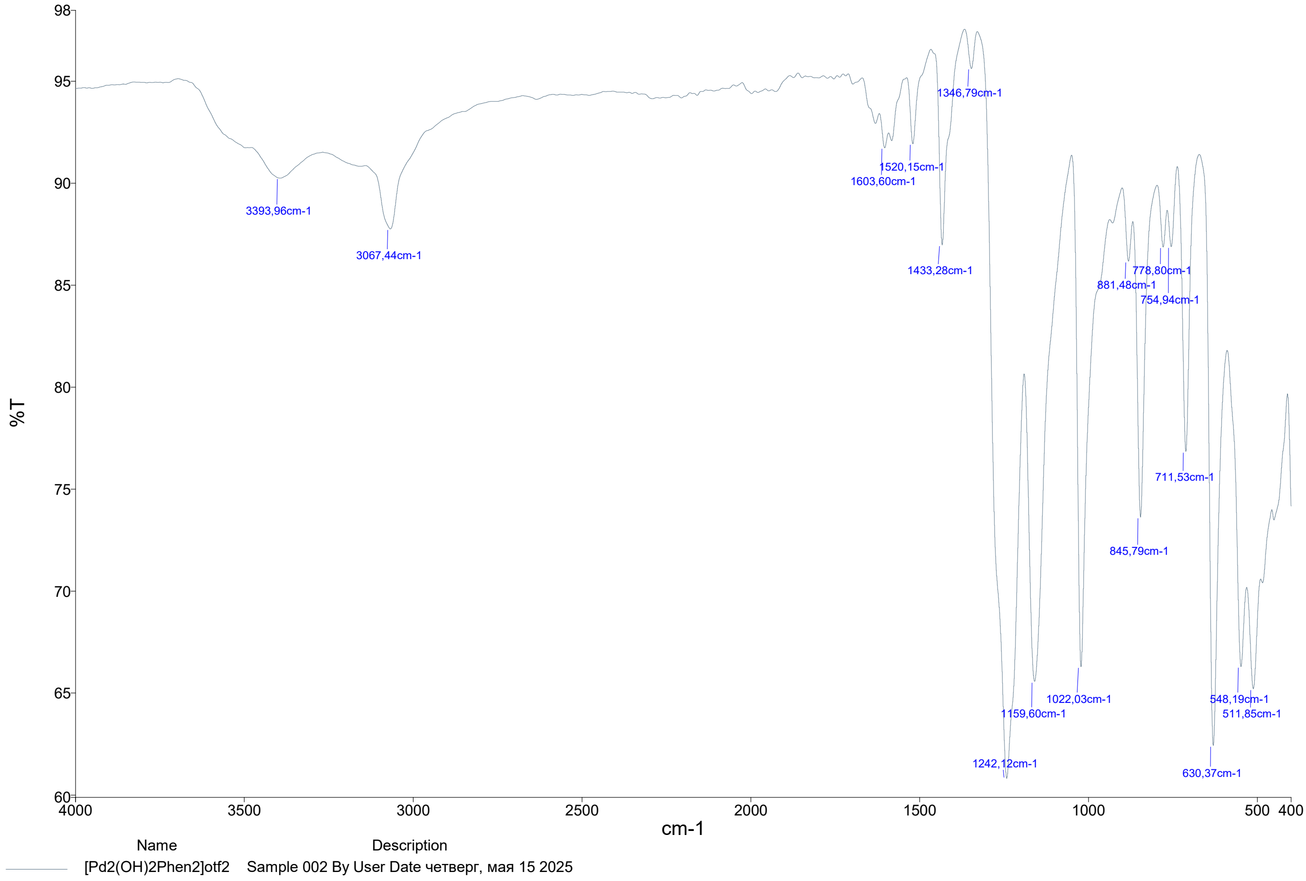


**Figure S4.** FTIR spectra of 3

$^1H$ NMR ($CD_3CN$, ppm): 8.73 (d. 2H, *J* = 9.2 Hz), 8.44 (d. 2H, *J* = 4.6 Hz), 8.09 (s. 2H), 7.92 (m. 2H), 1.19 (s. 18H).

**1**

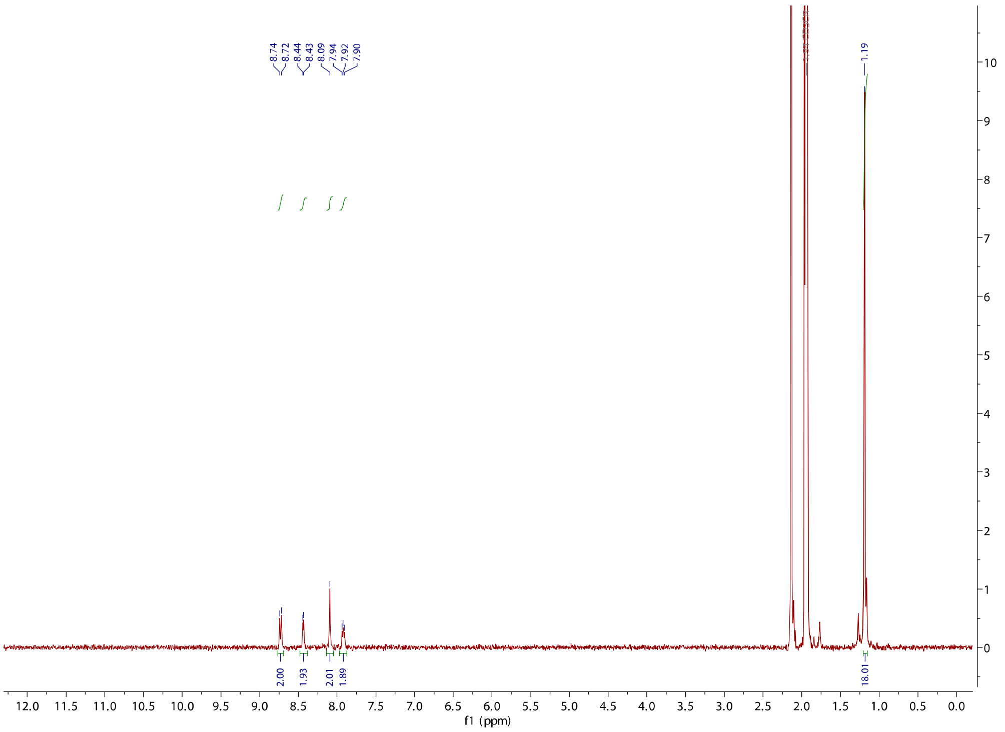


**2**

$^1H$ NMR ($CD_3CN$, ppm): 8.97 (dd. 2H, *J* = 8.3, 1.2 Hz), 8.85 (d. 2H, *J* = 5.5 Hz), 8.24 (s. 2H), 8.05 (dd. 2H, *J* = 8.3, 5.6 Hz).

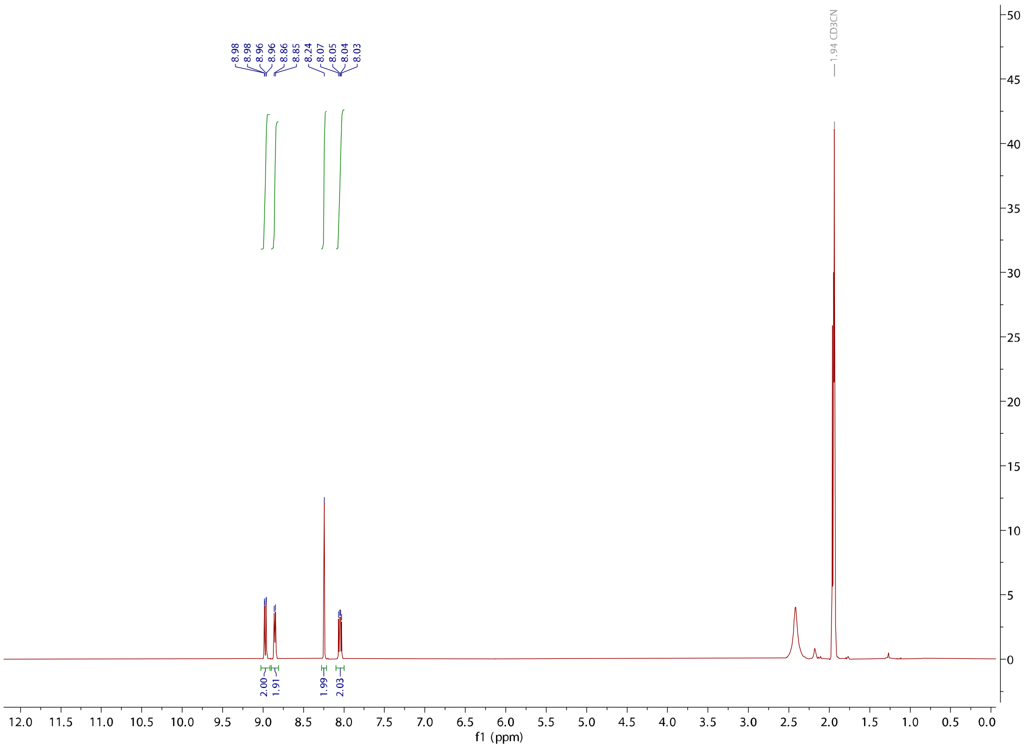


**Figure S5.** The $^1H$ NMR spectra of solutions compounds 1 and 2 in $CD_3CN$
